# Decompensated heart failure after one year of successful therapy with mavacamten: a case report

**DOI:** 10.1093/ehjcr/ytag741

**Published:** 2026-10-07

**Authors:** Giulia Marchionni, Lucrezia Netti, Silvia Stavagna, Cristina Chimenti

**Affiliations:** Department of Medical and Cardiovascular Sciences, Sapienza University of Rome, Viale del Policlinico 155, Rome 00161, Italy; Department of Medical and Cardiovascular Sciences, Sapienza University of Rome, Viale del Policlinico 155, Rome 00161, Italy; Department of Medical and Cardiovascular Sciences, Sapienza University of Rome, Viale del Policlinico 155, Rome 00161, Italy; Department of Medical and Cardiovascular Sciences, Sapienza University of Rome, Viale del Policlinico 155, Rome 00161, Italy

**Keywords:** Heart failure, Mavacamten, Drug–drug interaction, Systolic dysfunction, Hypertrophic cardiomyopathy, Case report

## Abstract

**Background:**

Cardiac myosin inhibition is the first disease-specific therapeutic strategy for obstructive hypertrophic cardiomyopathy. Mavacamten, the first-in-class agent, has demonstrated an excellent safety profile and sustained efficacy in both clinical trials and real-world practice. However, its safe use requires careful monitoring and thorough patient education regarding potential drug–drug interactions.

**Case summary:**

We report a case of acute heart failure occurring after long-term clinical stability on mavacamten following concomitant fluconazole administration. It is reasonable to assume that the pharmacokinetic interaction resulted in excessive mavacamten exposure, leading to transient systolic dysfunction, severe functional mitral regurgitation, and adverse left ventricular remodelling.

**Discussion:**

This case report highlights the consequences of pharmacological interactions between mavacamten and a strong CYP2C19 inhibitor. Drug–drug interaction should always be considered and systematically excluded in a patient presenting with unexplained heart failure and left ventricular remodelling during mavacamten therapy.

Learning points

- Concomitant administration of strong CYP2C19 inhibitors during mavacamten therapy can increase drug exposure and precipitate iatrogenic systolic dysfunction and acute heart failure.
- In patients receiving mavacamten who present with new-onset heart failure and adverse left ventricular remodelling, unrecognized drug–drug interactions should be systematically excluded.

## Background

Mavacamten, a first-in-class selective cardiac myosin inhibitor, has demonstrated extraordinary efficacy in improving symptoms, left ventricular outflow tract (LVOT) obstruction, and functional capacity in patients with obstructive hypertrophic cardiomyopathy (HCM).^1–4^ Its clinical benefit, however, is closely linked to precise dose titration and periodic monitoring of left ventricular systolic function. The drug is predominantly metabolized by hepatic cytochrome P450 enzymes, especially CYP2C19, making its pharmacokinetics susceptible to genetic variability and drug–drug interactions.^5^ Concomitant administration of strong CYP2C19 inhibitors can markedly increase mavacamten plasma concentration, predisposing patients to systolic dysfunction and heart failure.^6^ We describe a case of acute heart failure with adverse left ventricular remodelling occurring after more than one year of successful therapy with mavacamten triggered by unreported concomitant antimycotic therapy with fluconazole.

## Summary figure

**Figure:**
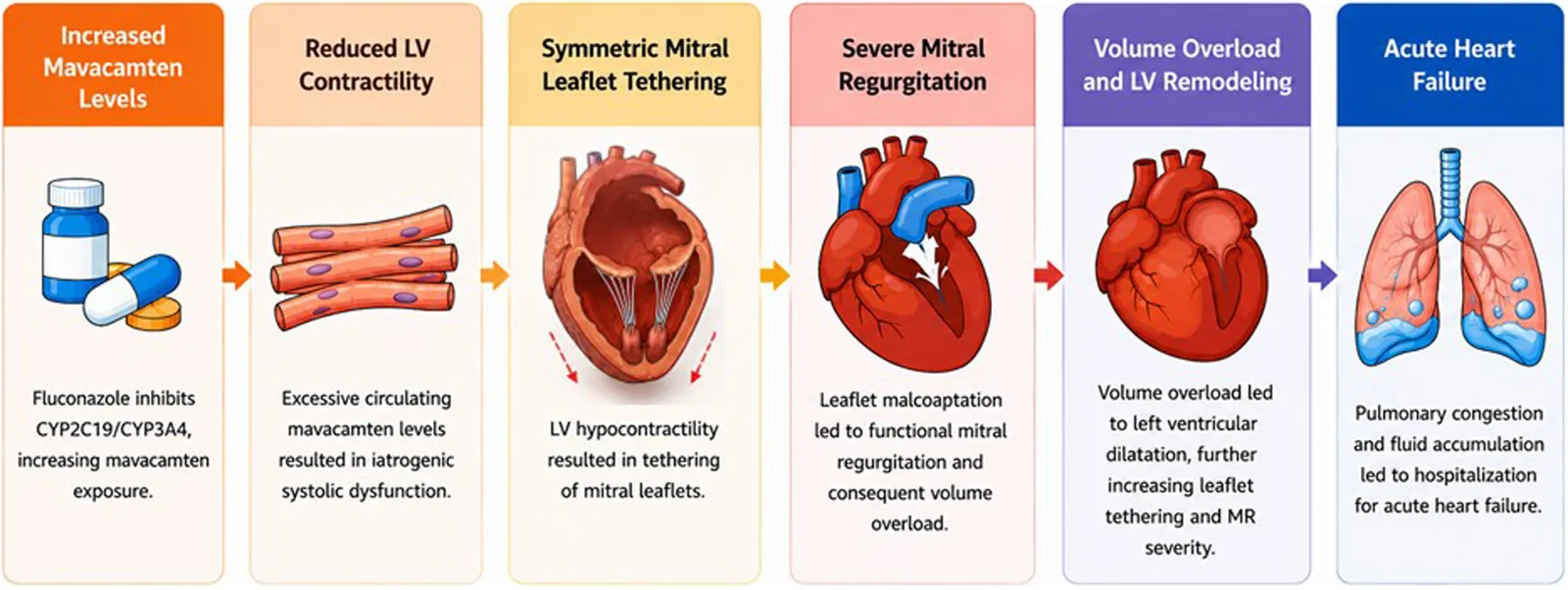


## Case presentation

In July 2025, a 65-year-old woman presented to the emergency department with progressively worsening dyspnoea over the preceding month. The patient started treatment with mavacamten in May 2024 for symptomatic obstructive HCM, which had been diagnosed in 2023 during a routine cardiological evaluation (see Supplementary material online, *Video S1*, *Figure 1*). Cardiac magnetic resonance performed at diagnosis demonstrated a maximal wall thickness of 20 mm at the basal anterior interventricular septum (IVS), a left ventricular (LV) mass of 65 g/m^2^, and small areas of non-ischaemic late gadolinium enhancement localized within the IVS. Her medical history was also notable for hypothyroidism following total thyroidectomy, for which she was receiving levothyroxine replacement therapy. For more than one year, the patient had been successfully treated with mavacamten at our centre, achieving complete resolution of symptoms and LVOT obstruction, with minimal residual mitral regurgitation (MR) and normal LV filling and pulmonary pressures.

**Figure 1:**
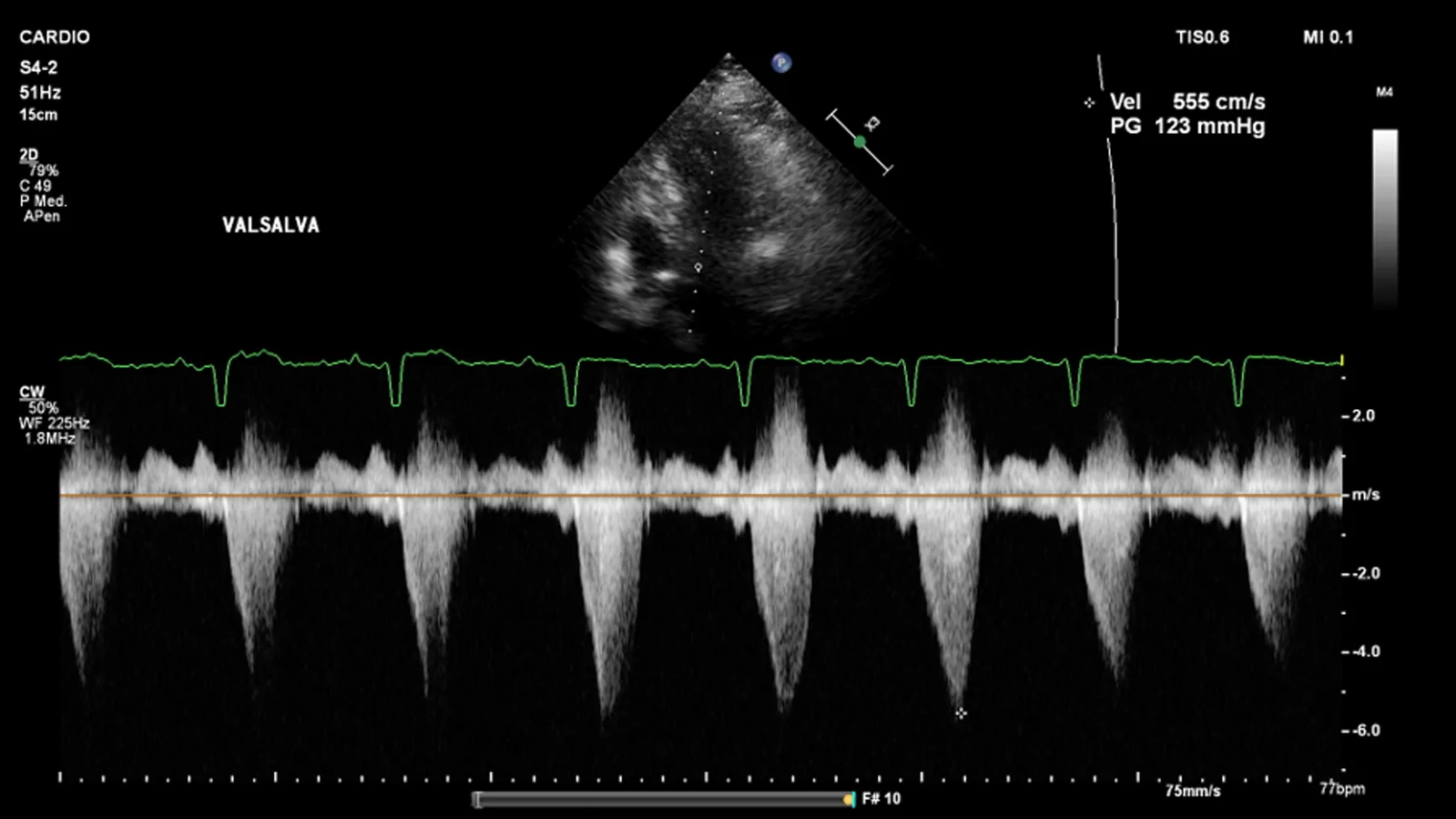
Continuous wave Doppler across left ventricular outflow tract showing left ventricular outflow tract gradient of 123 mmHg with Valsalva manoeuvre at diagnosis in 2023.

On admission, the patient was haemodynamically stable, afebrile, and in New York Heart Association (NYHA) functional Class III. Physical examination revealed reduced breath sounds bilaterally, bibasal crackles, and a grade 4/6 holosystolic murmur radiating to the left axilla. Chest computed tomography (*Figure 2*) demonstrated bilateral hilar congestion, pleural effusion, and findings consistent with possible lobar pneumonia. Transthoracic echocardiography showed no regional wall motion abnormalities and no LVOT obstruction. However, LV was dilated, with an indexed LV end-diastolic volume of 73 mL/m^2^ compared with 42 mL/m^2^ in April 2025, accompanied by a relative decline in left ventricular ejection fraction (LVEF) from 60% to 55% (see Supplementary material online, *Video S2*). Colour Doppler echocardiography demonstrated severe MR, predominantly secondary to symmetric leaflet tethering, with elevated LV filling pressures and pulmonary pressures (*Figure 3*). Laboratory tests showed normal troponin levels with complete blood cell count and inflammatory markers within normal limits. A detailed medical history revealed that the patient's clinical deterioration began shortly after an unreported 2-week course of fluconazole (250 mg/day), prescribed for cutaneous mycosis in May 2025. This temporal relationship strongly suggested iatrogenic systolic dysfunction secondary to a drug–drug interaction as the most likely cause of the presenting clinical picture. The patient was treated with intravenous loop diuretic therapy, while guideline-directed medical therapy for heart failure was optimized by increasing ramipril from 2.5 to 5 mg daily, initiating canrenone at 50 mg daily, and maintaining the SGLT2 inhibitor as part of her routine treatment. Although LVEF remained within the preserved range at presentation, it had declined compared with the stable value of 60% consistently documented during scheduled follow-up visits done in the last year while receiving mavacamten 10 mg daily. Therefore, the mavacamten dose was reduced from 10 to 5 mg daily to facilitate recovery while minimizing the risk of recurrent LVOT obstruction.

**Figure 2:**
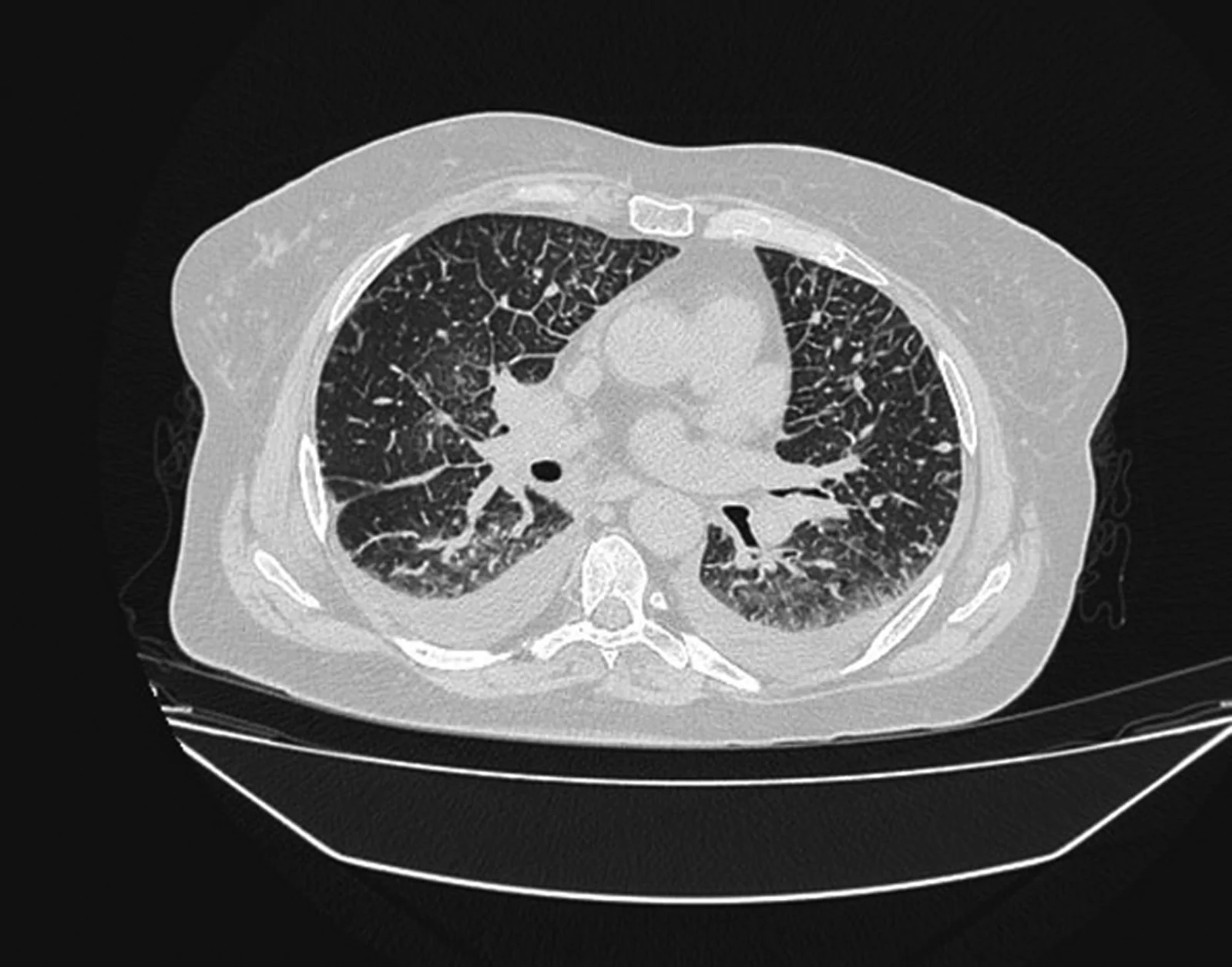
Chest computed tomography demonstrated features consistent with bilateral hilar congestion and pleural effusion.

**Figure 3:**
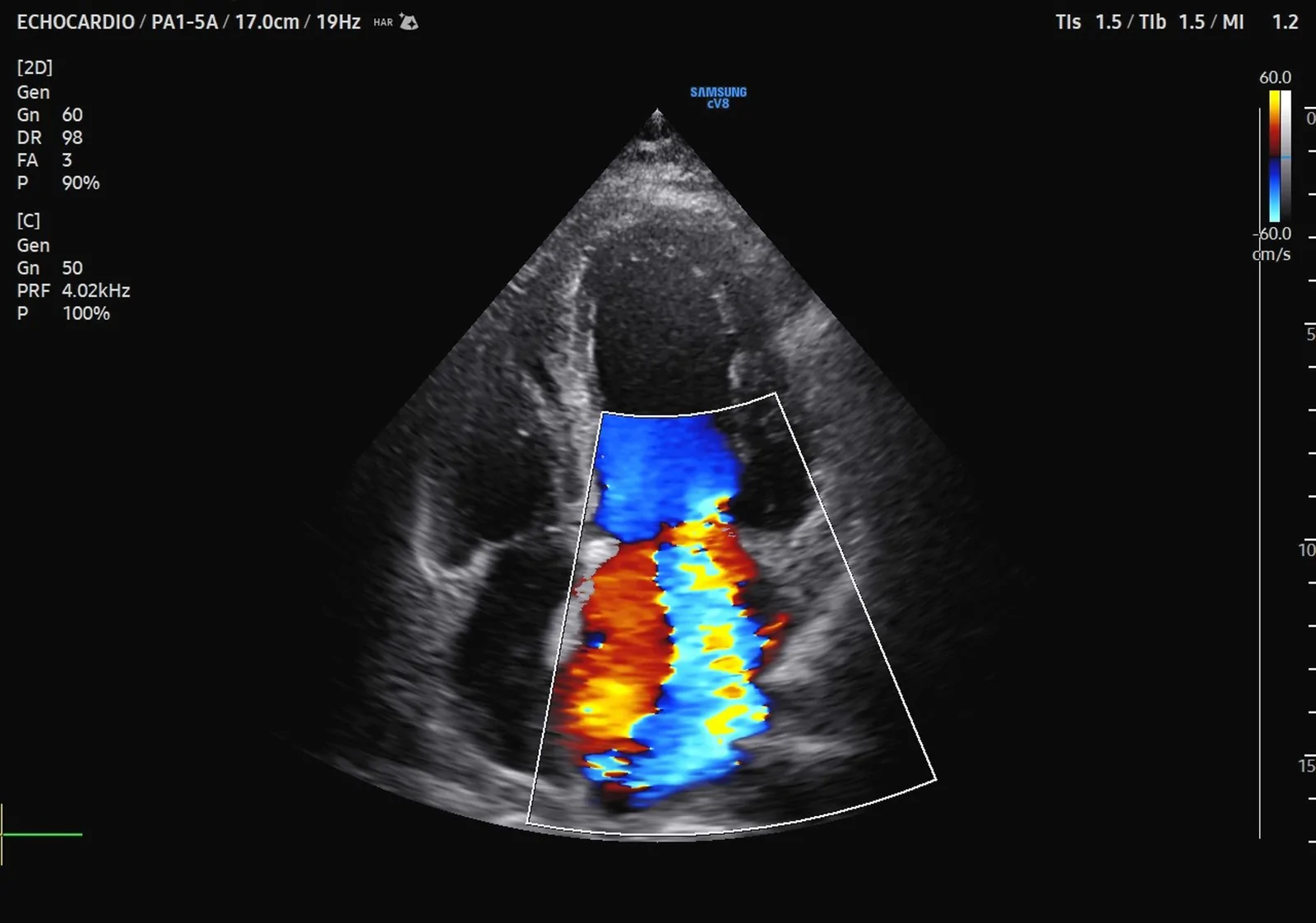
Apical four-chamber view at transthoracic echocardiography showing severe mitral regurgitation.

One month after hospital discharge, the patient reported a considerable improvement in her functional status, in NYHA Class II, without signs of central or peripheral congestion. Transthoracic echocardiography demonstrated a LVEF of 60%, mild MR (see Supplementary material online, *Videos S3* and *S4*), reduced left ventricular dimensions and normal LV filling pressures, with a resting LVOT gradient of 13 and 56 mmHg with Valsalva manoeuvre (*Figure 4*), overall conferring eligibility for Mavacamten uptitration to the previous 10 mg daily dose.

**Figure 4:**
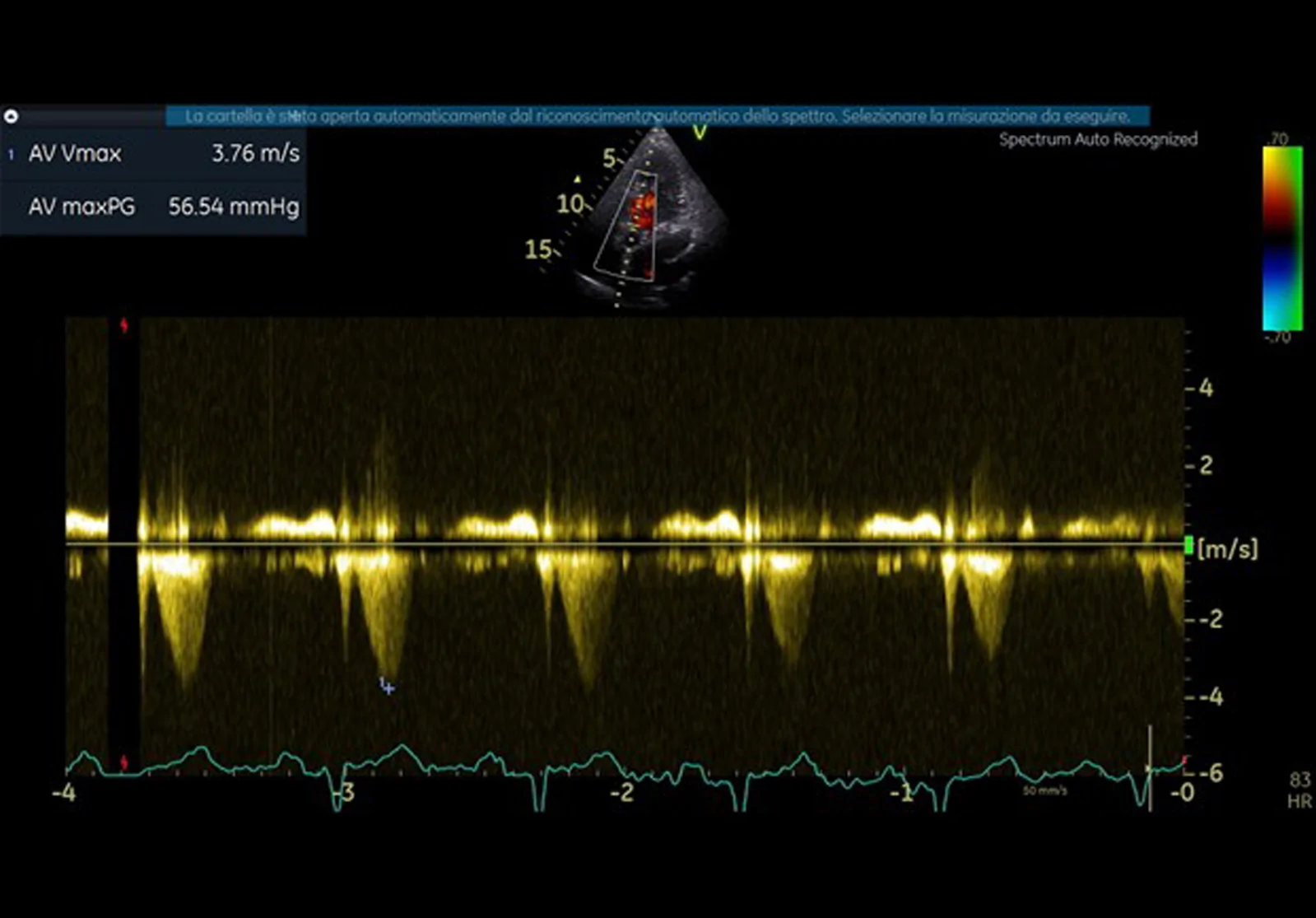
Continuous wave Doppler across left ventricular outflow tract showing left ventricular outflow tract gradient of 56 mmHg with Valsalva manoeuvre.

## Discussion

Mavacamten is extensively metabolized in the liver by cytochrome P450 enzymes, predominantly CYP2C19 (74%), CYP3A4 (18%), and CYP2C9 (8%).^5^ For this reason, patient’s CYP2C19 genotyping is mandatory to guide dosing and careful dose adjustment is essential when mavacamten is co-administered with cytochrome P450 modulators. Strong CYP2C19 inhibitors elevate plasma mavacamten levels and increase the risk of systolic dysfunction.^6^ Current recommendations advise either temporary discontinuation or dose reduction of mavacamten when co-administered with potent CYP2C19 inhibitors, along with close echocardiographic surveillance. Strong inhibitors of this enzyme, including fluconazole, substantially increase mavacamten bioavailability and prolong its half-life. Fluconazole, an antimycotic agent belonging to the triazole family, is a potent inhibitor of both CYP2C19 and CYP3A4^7^: current recommendations suggest that in a non-poor metabolizer already receiving mavacamten, concomitant fluconazole administration should be accompanied by mavacamten dose reduction to 2.5 mg/day, with follow-up assessment of LVEF after 4 weeks.^8^ In our patient, the 10 mg daily dose of mavacamten was inadvertently continued throughout the unreported course of fluconazole. The resulting increase in mavacamten exposure likely caused an excessive reduction in myocardial contractility. This, in turn, increased MR severity through symmetric leaflet tethering, resulting in persistent volume overload and progressive LV dilatation, which further exacerbated leaflet tethering and perpetuated the vicious cycle. According to the patient's history, symptoms began towards the end of concomitant fluconazole and mavacamten therapy and progressively worsened thereafter, in line with the prolonged half-life of mavacamten and the haemodynamic consequences of severe functional MR. The patient presented to our institution 1 month after completing the fluconazole course. Therefore, it is reasonable to assume that the preserved LVEF observed at admission reflected the progressive decline in circulating mavacamten concentrations following fluconazole withdrawal. Consequently, the clinical presentation represented the late phase of the unrecognized drug–drug interaction, with persistent LV dilatation and severe MR sustaining the presenting clinical picture despite partial recovery of systolic function. Although plasma mavacamten concentrations were not measured, excessive drug exposure was considered to have largely resolved by the time of reassessment, based on the recovery of LVEF and the known elimination half-life of mavacamten in CYP2C19 normal metabolizers, estimated to be around 9 days.^5^ For this reason, mavacamten was not interrupted but reduced to 5 mg daily. One month after hospital discharge, reverse LV remodelling and complete normalization of systolic function were accompanied by a marked reduction in MR severity, which returned to the mild residual degree documented in April 2025, further supporting the proposed pathophysiological mechanism.

## Conclusions

This clinical case describes the clinical consequences of an unreported concomitant administration of fluconazole and mavacamten in a patient with long-term optimal response to cardiac myosin inhibition. In this patient, the drug-induced reduction in systolic contractility induced severe MR and volume overload, ultimately leading to hospitalization for heart failure. As the use of cardiac myosin inhibitors expands, awareness of pharmacological interactions and patients’ education is crucial to ensure safety and maximize the therapeutic benefit of this extraordinary new therapeutic option for obstructive HCM.

## Lead author biography


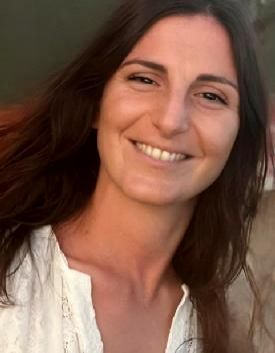


Giulia Marchionni graduated from Vita-Salute San Raffaele University in Milan in 2019 and completed her residency in Cardiology at the University of Pavia. She is currently enrolled in a joint PhD programme at Sapienza University of Rome and the University of Padua. Her clinical and research interests focus primarily on cardiomyopathies and inherited cardiac diseases.

## Supplementary Material

ytag741_Supplementary_Data

## Data Availability

The data underlying this article will be shared on reasonable request to the corresponding author.

## References

[1] Arbelo E, Protonotarios A, Gimeno JR, Arbustini E, Barriales-Villa R, Basso C, et al 2023 ESC guidelines for the management of cardiomyopathies: developed by the task force on the management of cardiomyopathies of the European Society of Cardiology (ESC). Eur Heart J 2023;44:3503–3626. 10.1093/eurheartj/ehad194 37622657

[2] Ommen SR, Ho CY, Asif IM, Balaji S, Burke MA, Day SM, et al 2024 AHA/ACC/AMSSM/HRS/PACES/SCMR guideline for the management of hypertrophic cardiomyopathy: a report of the American Heart Association/American College of Cardiology Joint Committee on clinical practice guidelines. Circulation 2024;149:e1239–e1311. 10.1161/CIR.0000000000001250 38718139

[3] Olivotto I, Oreziak A, Barriales-Villa R, Abraham TP, Masri A, Garcia-Pavia P, et al Mavacamten for treatment of symptomatic obstructive hypertrophic cardiomyopathy (EXPLORER-HCM): a randomised, double-blind, placebo-controlled, phase 3 trial. Lancet 2020;396:759–769. 10.1016/S0140-6736(20)31792-X 32871100

[4] Desai MY, Owens A, Wolski K, Geske JB, Saberi S, Wang A, et al Mavacamten in patients with hypertrophic cardiomyopathy referred for septal reduction: week 56 results from the VALOR-HCM randomized clinical trial. JAMA Cardiol 2023;8:968–977. 10.1001/jamacardio.2023.3342 PMC10463171 37639243

[5] Braunwald E, Saberi S, Abraham TP, Elliott PM, Olivotto I. Mavacamten: a first-in-class myosin inhibitor for obstructive hypertrophic cardiomyopathy. Eur Heart J 2023;44:4622–4633. 10.1093/eurheartj/ehad637 PMC10659958 37804245

[6] Klomp SD, Manson ML, Guchelaar HJ, Swen JJ. Phenoconversion of cytochrome P450 metabolism: a systematic review. J Clin Med 2020;9:2890. 10.3390/jcm9092890 PMC7565093 32906709

[7] Niwa T, Shiraga T, Takagi A. Effect of antifungal drugs on cytochrome P450 (CYP) 2C9, CYP2C19, and CYP3A4 activities in human liver microsomes. Biol Pharm Bull 2005;28:1805–1808. 10.1248/bpb.28.1805 16141567

[8] Ricci F, Molinari LV, Mansour D, Galanti K, Vagnarelli F, Renda G, et al Managing drug–drug interactions with mavacamten: a focus on combined use of antiarrhythmic drugs and anticoagulants. Heart Rhythm 2025;22:510–525. 10.1016/j.hrthm.2024.11.041 39613202

